# Foreign Summary

**Published:** 1840-02-29

**Authors:** 


### FOREIGN SUMMARY.

*Singular Pathological Condition of the Left Pleura.*—At a meeting of the Westminster Medical Society, Dr. Golding Bird exhibited a pathological specimen, curious from its anomalous character, rather than from its practical importance. The patient from whom it was taken died in half an hour after his admission into Guy's Hospital, in a sinking state. No previous history of the case was obtained; but the patient had a general bloated appearance, his lips were livid, he was cold, and in a state of collapse. A small quantity of urine which he made was found to be albuminous. It was suspected that he was perishing from the disease known under the name of "Bright's kidney." The body was examined about thirty-six hours after death. The kidneys were found to be in the first stage of the suspected disease. The heart was somewhat hypertrophied; the sigmoid valves slightly ossified. The lungs afforded an admirable specimen of pulmonary apoplexy, being saturated with blood. On cutting open the left cavity of the pleura, a curious body was observed, pushing that membrane forwards, and projecting into the cavity of the chest, exactly like a number of pig's teats. On examining them, they bore a greater resemblance to them than any thing else. They proved to be of a steatomatous character; and it appeared, on further examination, that a steatomatous tumour having formed in the anterior mediastinum, had pushed the pleura of the left side forward, and produced the singular appearance observed.

*London Lancet.*

